# P-1078. Predictive factors of bloodstream infections caused by carbapenem-resistant Acinetobacter baumannii in critically ill patients with any site colonization: a prospective observational study

**DOI:** 10.1093/ofid/ofaf695.1273

**Published:** 2026-01-11

**Authors:** Giusy Tiseo, Valentina Galfo, Aurelio Lepore, Lorenzo Suardi, Manuela Pogliaghi, Marco Falcone

**Affiliations:** Infectious Diseases Unit/University of Pisa, Pisa, Toscana, Italy; University of Pisa, Pisa, Toscana, Italy; University of Pisa, Pisa, Toscana, Italy; University Hospital of Pisa, Pisa, Toscana, Italy; University Hospital of Pisa, Pisa, Toscana, Italy; Infectious Diseases Unit/University of Pisa, Pisa, Toscana, Italy

## Abstract

**Background:**

In patients with bloodstream infections (BSIs) caused by carbapenem-resistant *Acinetobacter baumannii* (CRAB) the delay in appropriate antibiotic therapy is associated with high mortality risk. Our aim was to assess the risk of BSI by CRAB in patients with CRAB colonization and to develop a predictive score for CRAB BSI

**Methods:**

Observational, prospective study including consecutive adult patients admitted to intensive care unit (ICU) colonized by CRAB at any anatomical site at the University Hospital of Pisa, Italy (2020-2023). A systematic surveillance with rectal swab twice weekly and screening of other sites (urine, respiratory tract samples, skin) once weekly was implemented. The primary outcome was the occurrence of BSI by CRAB. A multivariable regression analysis was performed to identify factors associated with CRAB-BSI. Regression coefficients were used to develop the score. Discrimination was evaluated using the area under the ROC curve (AUC).

**Results:**

Among 283 patients colonized by CRAB, the rate of BSI by CRAB was 36.4% (103/283). Comparison of patients who developed CRAB BSI and those who did not is shown in Table 1. On multivariable analysis, burns (OR 8.219, 95% CI 3.591-18.812, p< 0.001), number of colonized sites (per site: OR 2.197, 95%CI 1.363-3.541, p=0.001), respiratory tract colonization (OR 4.285, 95%CI 2.179-8.426, p< 0.001), and cardiovascular disease (OR 1.940, 95%CI 1.068-3.524, p=0.029) were independently associated with risk of CRAB BSI. The risk score is reported in Table 2. The AUC of the model was 0.817 (95% CI 0.764-0.869, p < 0.001). The goodness of fit Hosmer-Lemeshow test showed a good calibration (χ²=5.068, p=0.535). As shown in Figure 1, the negative predictive value (NPV) was 72.2% in patients who developed an episode of septic shock during hospitalization and 97.8% in those without septic shock.

**Conclusion:**

This score can be useful for a rational empirical use of new antibiotics. In ICU patients colonized by CRAB, empirical therapy covering CRAB should be considered if septic shock occurs independently by the score. Conversely, in patients without septic shock and a negative score (< 2), the risk of CRAB BSI was low and empirical use of new anti-CRAB antibiotics may be avoided.

**Disclosures:**

Giusy Tiseo, MD, MSD, Gileal, Menarini, Advanz Pharma: Honoraria Marco Falcone, MD, PhD, Pfizer, Menarini, MSD, Gilead, Shionogi: Board Member|Pfizer, Menarini, MSD, Gilead, Shionogi: Grant/Research Support|Pfizer, Menarini, MSD, Gilead, Shionogi: Honoraria

